# Association of omega-3 fatty acids with improved prognosis after myocardial infarction: the role of red cell distribution width—an EHR study

**DOI:** 10.1186/s40001-026-03909-9

**Published:** 2026-01-21

**Authors:** Yu Wang, Caixia Dong, Yanjing Feng, Wenqian Zhang, Miao Yuan, Yao Ma, Chang Liu, Dengfeng Gao

**Affiliations:** 1 Department of Cardiology, The Second Affiliated Hospital of Xi’an Jiaotong University, NO.157, West 5th Road, Xi’an, 710004 Shaanxi China; 2 National-Local Joint Engineering Research Center of Biodiagnosis & Biotherapy, The Second Affiliated Hospital of Xi’an Jiaotong University, NO.157, West 5th Road, Xi’an, 710004 Shaanxi China

**Keywords:** Omega-3 fatty acid, Red cell distribution width, Myocardial infarction, Prognosis, Electronic health record

## Abstract

**Background:**

A high red cell distribution width (RDW) reflects an inflammatory state and abnormal red blood cell function, which has been associated with poor prognosis in patients with prior myocardial infarction (MI). Omega-3 fatty acids (n-3 FAs) are inversely related to RDW in the general population but have not been studied in post-MI patients. We aimed to explore associations between n-3 FAs, RDW and prognosis in individuals with prior MI by analyzing electronic health records (EHRs).

**Methods:**

This study analyzed two cohorts from the National Health and Nutrition Examination Survey (NHANES) and the UK Biobank databases. In the NHANES cohort, associations between dietary n-3 FA intake, RDW, and mortality were explored using multivariable Cox proportional hazards regression and restricted cubic splines (RCSs). In the UK Biobank cohort, associations between n-3 blood biomarkers, RDW, and 5 year mortality were examined through Spearman correlations, multivariate Cox proportional hazards regression, RCSs, and Karlson–Holm–Breen (KHB) mediation analysis. Finally, in the internal dataset, this study explored the potential role of RDW in the association between plasma eicosapentaenoic acid (EPA) level and the 6 month major adverse cardiovascular events (MACE) incidence using KHB mediation analysis.

**Results:**

RDW was positively associated with mortality in both cohorts from public databases. Dietary n-3 FA intake were inversely associated with RDW. Blood n-3 biomarkers were significantly inversely associated with RDW and 5 year mortality. Moreover, RDW appears to partially mediate the relationship between n-3 blood biomarkers and 5 year mortality. The internal dataset also suggested that RDW may modestly influence the association between plasma EPA level and 6-month MACE incidence.

**Conclusions:**

Both dietary and blood levels of n-3 FAs are negatively associated with RDW in individuals with prior MI. The findings are consistent with the hypothesis that RDW may play a potential mediating role in the association between n-3 FAs and mortality risk. Given that a high RDW signifies an inflammatory state and abnormal erythrocyte function, the observed association between higher blood levels of n-3 FAs and improved prognosis after MI aligns with potential underlying mechanisms: attenuated inflammation and enhanced erythrocyte function.

**Supplementary Information:**

The online version contains supplementary material available at 10.1186/s40001-026-03909-9.

## Background

Acute myocardial infarction (MI) is a leading cause of death worldwide and imposes a substantial health burden [1]. Although acute-phase mortality has declined owing to advancements in percutaneous coronary interventions, intermediate- and long-term mortality remains elevated compared to the general population [2]. Red cell distribution width (RDW) has emerged as a key prognostic biomarker for MI [3]. Consistent with this, our previous study [4] developed a neural network model to predict MI prognosis using complete blood count and differentials, in which RDW emerged as one of the top three predictors. The prognostic value of RDW is well established, with underlying biological mechanisms potentially involving functional alterations in erythrocytes under ischemic and hypoxic conditions—thereby exacerbating cardiac injury [5]—as well as RDW’s function as an indicator of systemic inflammation [6]. Post-MI inflammation can lead to adverse remodeling, contributing to heart failure development and increased mortality [7]. Omega-3 fatty acids (n-3 FAs) offer multiple effects, such as attenuating inflammation and enhancing cellular function [8]. Nevertheless, the associations between n-3 FAs and RDW in post-MI individuals remain unexplored.

Although n-3 FAs are widely recognized for their cardioprotective benefits, recent large-scale clinical trials have reported inconsistent results [9–11]. Notably, randomized trials specifically enrolling patients after MI have largely yielded neutral findings. For instance, the OMEMI trial [12] randomized 1014 elderly patients within 2–8 weeks post-MI to 1.8 g/day of n-3 FAs or placebo and observed no significant reduction in major adverse cardiovascular events (MACE) over 2 years. Similarly, earlier secondary prevention trials—such as Alpha Omega [13] and GISSI-Prevenzione [14]—demonstrated inconsistent or modest benefits. These inconsistent findings underscore the complexity of translating n-3 supplementation into clinical benefit in high-risk post-MI populations and highlight the need to identify biological pathways through which n-3 FAs may still exert protective effects. Recent studies have investigated the distinct mechanisms of action of eicosapentaenoic acid (EPA) and docosahexaenoic acid (DHA) [15–17], providing partial explanations for these discrepancies. Nonetheless, further research is essential to fully elucidate these differences.

Icosapent ethyl (IPE), a highly purified derivative of EPA, has been incorporated into numerous authoritative guidelines. These guidelines primarily recommend IPE for patients with atherosclerotic cardiovascular disease who continue to exhibit elevated triglyceride (TG) levels despite treatment with the maximum tolerated dose of statins. However, findings from key n-3 FAs trials reported to date have shown no clear correlation between TG lowering and cardiovascular events [18]. Consequently, conducting comprehensive further studies to elucidate the mechanisms of EPA that extend beyond TG reduction is essential.

Based on the foregoing evidence, we hypothesize a plausible biological link between n-3 FAs and outcomes post-MI, potentially mediated by anti-inflammatory and erythrocyte-stabilizing effects that may be reflected in hematologic markers such as RDW. However, it remains unclear whether the association between n-3 FAs and RDW persists in individuals following MI, which specific n-3 FA component confers the predominant cardiovascular benefit, and whether RDW may partially account for the association between n-3 FAs and prognosis in this high-risk population.

In this study, we investigated how dietary intake of EPA and DHA, along with levels of n-3 blood biomarkers, are related to RDW and mortality following MI in two cohorts from public databases. We then addressed a similar question in an internal prospective cohort, focusing specifically on the associations between plasma EPA levels, RDW measured during the acute phase, and the incidence of MACE at 6 months. Given that 6-month MACE is strongly linked to poor intermediate- and long-term prognosis, we anticipate that findings from our internal cohort will provide supportive evidence for the associations observed in the public datasets. Ultimately, we aim to raise clinical awareness of RDW as a prognostic indicator and to advance understanding of the potential mechanisms underlying the cardioprotective effects of n-3 FAs.

## Methods

### Study design, data source and ethical statement

We first conducted retrospective cohort studies using two publicly available datasets. Subsequently, we addressed a similar question in a prospective internal dataset collected by our research team.

The National Health and Nutrition Examination Survey (NHANES) is a nationally representative cross-sectional survey in the United States that assesses the health and nutritional status of adults and children (https://wwwn.cdc.gov/nchs/nhanes/default.aspx). It enables linkage with the Centers for Disease Control's National Death Index to obtain mortality outcomes for survey participants (https://www.cdc.gov/nchs/data-linkage/mortality-public.htm). Using electronic health records (EHRs) derived from this database, we analyzed associations between dietary intake of EPA and DHA, RDW, and mortality following MI.

The UK Biobank is a large-scale cohort study comprising half a million participants, established to support research into the genetic, lifestyle, and environmental determinants of a wide spectrum of diseases. Leveraging EHRs sourced from the UK Biobank (http://www.ukbiobank.ac.uk), we examined the associations between n-3 blood biomarkers, RDW, and 5 year mortality following MI.

Both databases are publicly accessible and have received prior ethical approvals. The NHANES was approved by the National Center for Health Statistics’ Ethics Review Board, and the UK Biobank received approval from the National Research Ethics Service Committee of North West–Haydock. In both databases, participants provided informed consent, and all data are fully de-identified. Consequently, our institutional ethics committee waived the requirement for additional ethical review.

Moreover, we prospectively recruited patients with acute MI from the Department of Cardiology at the Second Affiliated Hospital of Xi’an Jiaotong University. All participants received comprehensive information regarding the study procedures prior to enrollment and provided written informed consent, authorizing the collection of blood samples and clinical data during their hospitalization. The study was conducted in accordance with the principles of the Declaration of Helsinki to ensure the full protection of patients’ rights and privacy. The research protocol was reviewed and approved by the hospital’s Medical Ethics Committee (Ethical Approval Document No. 2024–180).

### Study population

**Fig. 1:**
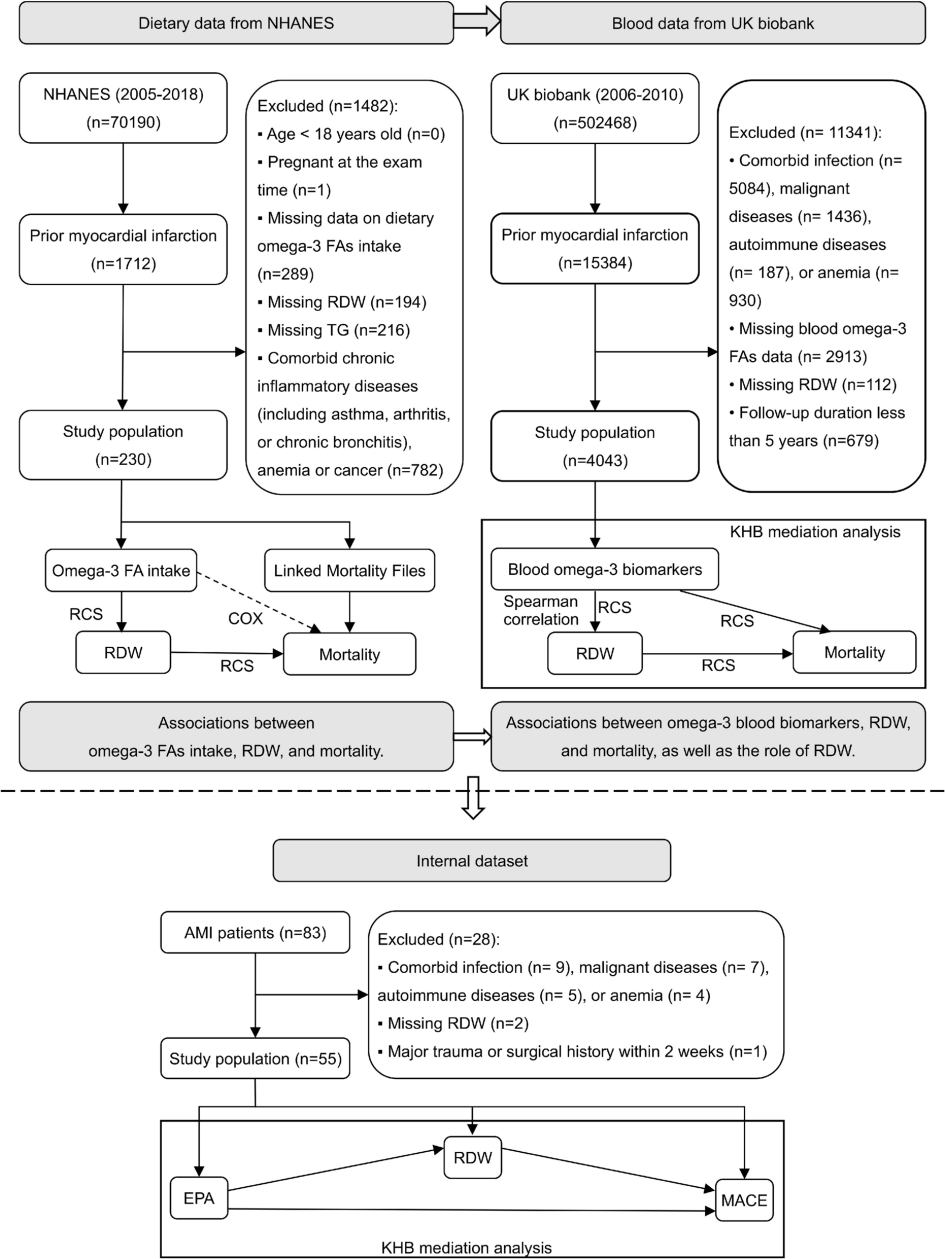
The research workflow of this study. Dashed arrow signifies the finding that failed to reach statistical significance. NHANES, the National Health and Nutrition Examination Survey; RDW, red cell distribution width—coefficient of variation; *TG* triglycerides, *RCS* restricted cubic spline, *FA* fatty acid, *EPA* eicosapentaenoic acid, *MACE* major adverse cardiovascular events

The flowchart of the study population selection is presented in Fig. 1.

In the NHANES database, we analyzed data from seven survey cycles spanning 2005 to 2018. Participants who responded "Yes" to the question MCQ160e (‘‘Ever told you had a heart attack?’’) were classified as having a prior MI. We excluded individuals younger than 18 years, pregnant women, and those with missing data on dietary n-3 FAs, RDW or TG, as well as those reporting comorbidities. These comorbidities included chronic inflammatory conditions (such as asthma, arthritis, or chronic bronchitis), anemia, and cancer. Pregnancy was defined based on both self-reported status and laboratory-confirmed indicators. Comorbidities were primarily ascertained through self-reported medical history; anemia was further confirmed using laboratory-measured hemoglobin levels (< 130 g/L in men and < 120 g/L in women [19]).

In the UK Biobank database, we identified 15,384 participants with a history of MI from 2006 to 2010. We excluded individuals with comorbid infections, malignant diseases, autoimmune diseases, or anemia, as well as those lacking data on n-3 blood biomarkers or RDW. Medical history was ascertained using International Classification of Diseases, 10th Revision codes. Additionally, participants with a follow-up duration of less than 5 years were excluded.

In the internal dataset, no formal sample size calculation was performed due to the high cost of blood FA testing and the exploratory nature of this pilot study. The final study population was determined based on predefined inclusion and exclusion criteria, patients’ hospitalization status, and the availability of informed consent. Hospitalized patients were prospectively and consecutively enrolled between August 2024 and December 2024. Eligible participants were those whose primary admission diagnosis was acute MI, including both ST-segment elevation MI and non-ST-segment elevation MI. Patients were excluded if they had acute or chronic infections, malignancies (including hematologic cancers), anemia (defined as hemoglobin < 130 g/L in men or < 120 g/L in women), or autoimmune diseases (such as rheumatoid arthritis, asthma, or chronic bronchitis). Additional exclusion criteria included a history of major trauma or surgery within 2 weeks, as well as missing RDW measurements at the time of admission.

### Variables

In the NHANES cohort, the primary variables of interest were dietary intake of EPA and DHA and RDW. Additionally, a set of covariates—prespecified based on established biological and clinical knowledge—was included in the analyses: age, sex, race, poverty-to-income ratio (PIR), body mass index (BMI), high-density lipoprotein cholesterol (HDL-C), total cholesterol, TG, low-density lipoprotein cholesterol (LDL-C), alcohol consumption status, smoking status, total energy intake, and comorbidities including hypertension, diabetes, kidney disease, congestive heart failure, and stroke. Dietary nutrient intake was estimated by averaging reported values from the first and second 24-h dietary recall interviews; if data were available for only one day, that single-day value was used. Alcohol and smoking status were classified based on responses to two specific questionnaire items. Those who answered “Never in the last year” to question ALQ121 (“Past 12 mo how often have alcohol drink”) were categorized as non–alcohol consumers. Those who responded “No” to question SMQ020 (“Smoked at least 100 cigarettes in life”) were classified as nonsmokers. All other responses were considered indicative of current or past alcohol consumption or smoking. Comorbidities were identified based on self-reported physician diagnoses from questionnaire data. Specifically, diabetes was defined as either a self-reported diagnosis or a glycated hemoglobin A1c level greater than 6.5% [20]. The use of antidiabetic medications was not incorporated into this definition.

In the UK Biobank cohort, our primary focus was on n-3 blood biomarkers and RDW. All n-3 blood biomarkers were measured from participants’ EDTA plasma samples using the high-throughput, nuclear magnetic resonance-based metabolic biomarker profiling platform developed by Nightingale Health Ltd. This platform provided several key metrics: “Field 23,444 omega-3 fatty acids” (referred to uniformly as n-3FA in subsequent analyses), representing the absolute concentration (in mmol/L) of all n-3 FAs; “Field 23,451 omega-3 fatty acids to total fatty acids percentage” (n-3FAp), reflecting the proportion (%) of total n-3 FAs relative to the sum of all measured FAs; “Field 23,450 DHA”, indicating the absolute concentration (in mmol/L) of DHA; and “Field 23,457 DHA to total fatty acids percentage” (DHAp), denoting the proportion (%) of DHA relative to total FAs. Additionally, we derived an estimated EPA to total FAs percentage (eEPAp) by subtracting DHAp from n-3FAp (eEPAp = n-3FAp − DHAp), a metric previously validated as a reliable proxy for the proportion (%) of EPA relative to total FAs [21]. We further calculated the EPA-to-DHA ratio (EDR) as the quotient of eEPAp divided by DHAp (EDR = eEPAp/DHAp). In addition to these biomarkers, covariates included age, sex, BMI, systolic blood pressure (SBP), diastolic blood pressure (DBP), smoking status, C-reactive protein (CRP), total cholesterol, creatinine, glucose, HDL-C, TG, and comorbidities such as atrial fibrillation, history of cardiac arrest, arrhythmia, hypertension, congestive heart failure, and valvular disease. Information on cardiovascular-related medications—including metformin, aspirin, clopidogrel, statins, β-blockers, angiotensin-converting enzyme inhibitors and angiotensin II receptor blockers (ACEIs/ARBs), and calcium channel blockers (CCBs)—was also incorporated as covariates.

In the internal dataset, 3–5 mL of peripheral venous blood was collected from each patient upon admission using EDTA-K₂ anticoagulant tubes. The samples were immediately centrifuged at 4 °C for 15 min at a speed of 2500–3000 rpm. Approximately 300 µL of plasma was then transferred into microcentrifuge tubes and stored at − 80 °C. All samples were subsequently shipped on dry ice to CTi Life Research Centre Co., Ltd. (Shanghai) for quantification of EPA using liquid chromatography–tandem mass spectrometry. Concurrently, baseline clinical data were systematically collected, including RDW, demographic characteristics (age and sex), vital signs (SBP and heart rate), comorbidities (such as hypertension [22] and diabetes[20]), smoking status, and a panel of biochemical laboratory parameters. The occurrence of MACE—defined as a composite of all-cause death, non-fatal MI, non-fatal stroke, and unplanned coronary revascularization—within six months was ascertained through structured telephone follow-up interviews.

In this study, the primary variables and outcome data were fully observed, with no missing values. For each covariate, the proportion of missing data was kept below 25%. In the NHANES cohort, missing values were imputed using the median of the corresponding variable within the same two-year survey cycle. In the UK Biobank cohort, missing data were addressed through multiple imputation by chained equations (MICE) prior to subsequent analyses.

### Statistical analysis

#### Weight selection and calculation in the NHANES cohort

We selected survey weights in accordance with the NHANES database guidelines. Specifically, we applied WTDR2D for participants who provided dietary intake data for both the first and second 24 h recall days, and WTDRD1 for those with data from the first day only. For the pooled analysis across seven two-year survey cycles, sample weights were derived by dividing the original 2 year cycle-specific weights by seven.

### Analysis of baseline characteristics

We assessed the normality of continuous variables using the Kolmogorov–Smirnov test. All continuous variables in this study were non-normally distributed and are therefore presented as medians with interquartile ranges. Group comparisons for continuous data were conducted using the Mann–Whitney U test, while categorical variables—expressed as frequencies and percentages—were compared using the Chi-square test.

### Studies on dietary n-3 FAs

To investigate the associations between dietary n-3 FAs (EPA and DHA) and RDW, we employed restricted cubic splines (RCSs) based on linear regression models, adjusting for all covariables. To examine the association between RDW and mortality, we applied RCSs derived from Cox regression models. Three models were considered: Model 0, unadjusted; Model 1, adjusted for age, sex, and race; and Model 2, further adjusted for all covariates. In all RCS analyses, three knots were placed at the 10th, 50th, and 90th percentiles of the exposure distribution.

To explore the associations between dietary n-3 FAs and mortality, we employed Cox models. Three models were fitted: Model 0, unadjusted; Model 1, adjusted for age, sex, and race; and Model 2, adjusted for all covariates. Model 2 was further stratified by LDL-C levels (2.6 mmol/L), total cholesterol levels (5.2 mmol/L), and alcohol consumption status. The proportional hazards assumption was assessed using Schoenfeld residuals tests; a *p* < 0.05 for any individual variable or for the global test was considered indicative of a potential violation.

### Studies on n-3 blood biomarkers

First, in the absence of a standard clinical threshold for RDW and to achieve balanced group sizes, we used the cohort-specific median RDW value (13.4%) as a cut-off to dichotomize the UK Biobank cohort into high- and low-RDW groups, and compared their baseline characteristics. Next, to investigate the associations between n-3 blood biomarkers and RDW, we generated scatter plots with fitted linear regression lines and performed Spearman correlation analyses, given the non-normal distribution of the data. To explore potential nonlinear relationships, we also constructed RCSs. Additionally, after standardizing each biomarker, we reported the change in RDW associated with a one-standard-deviation (SD) increase in that biomarker. We then fitted multivariable Cox models, incorporating RCSs to flexibly assess the associations between n-3 biomarkers and 5 year mortality. These exposure variables were standardized, and effect estimates are presented per SD increase. Proportional hazards diagnostics were performed. Variables exhibiting clear violations of proportionality were handled by stratification. All models were adjusted for the full set of prespecified covariates and further stratified by creatinine levels (100 μmol/L), glucose levels (11 mmol/L), atrial fibrillation, history of cardiac arrest, and statin use. Finally, we applied Karlson–Holm–Breen (KHB) mediation analysis [23, 24] within a logistic regression framework to evaluate the potential role of RDW in the associations between n-3 blood biomarkers and 5-year mortality. The outcome “5 year mortality” was defined as a binary variable indicating whether all-cause death occurred within five years of baseline (yes = 1, no = 0), with the time origin set as the date of MI diagnosis recorded in the UK Biobank database.

### Analysis of the internal dataset

We employed KHB mediation analysis to assess the potential role of RDW in the association between plasma EPA level and the incidence of 6-month MACE following MI. However, due to the limited sample size, statistical power was insufficient to support multivariable adjustment. Consequently, we did not adjust for potential confounders in this analysis.

The research workflow is illustrated in Fig. 1. Most statistical analyses were conducted using R software (version 4.4.0) [25] with commonly used packages. For the NHANES cohort, we employed the ‘nhanesA’ [26] and ‘survey’ [27] packages to properly account for the complex survey sampling design. KHB mediation analysis was performed using StataMP (Version 17; StataCorp LLC, 2021) [28]. All statistical tests were two-sided, and a *p*-value < 0.05 was considered statistically significant.

## Results

### Associations between dietary n-3 FA intake, RDW, and mortality following MI

#### Dietary n-3 FA intake was inversely associated with RDW

**Fig. 2:**
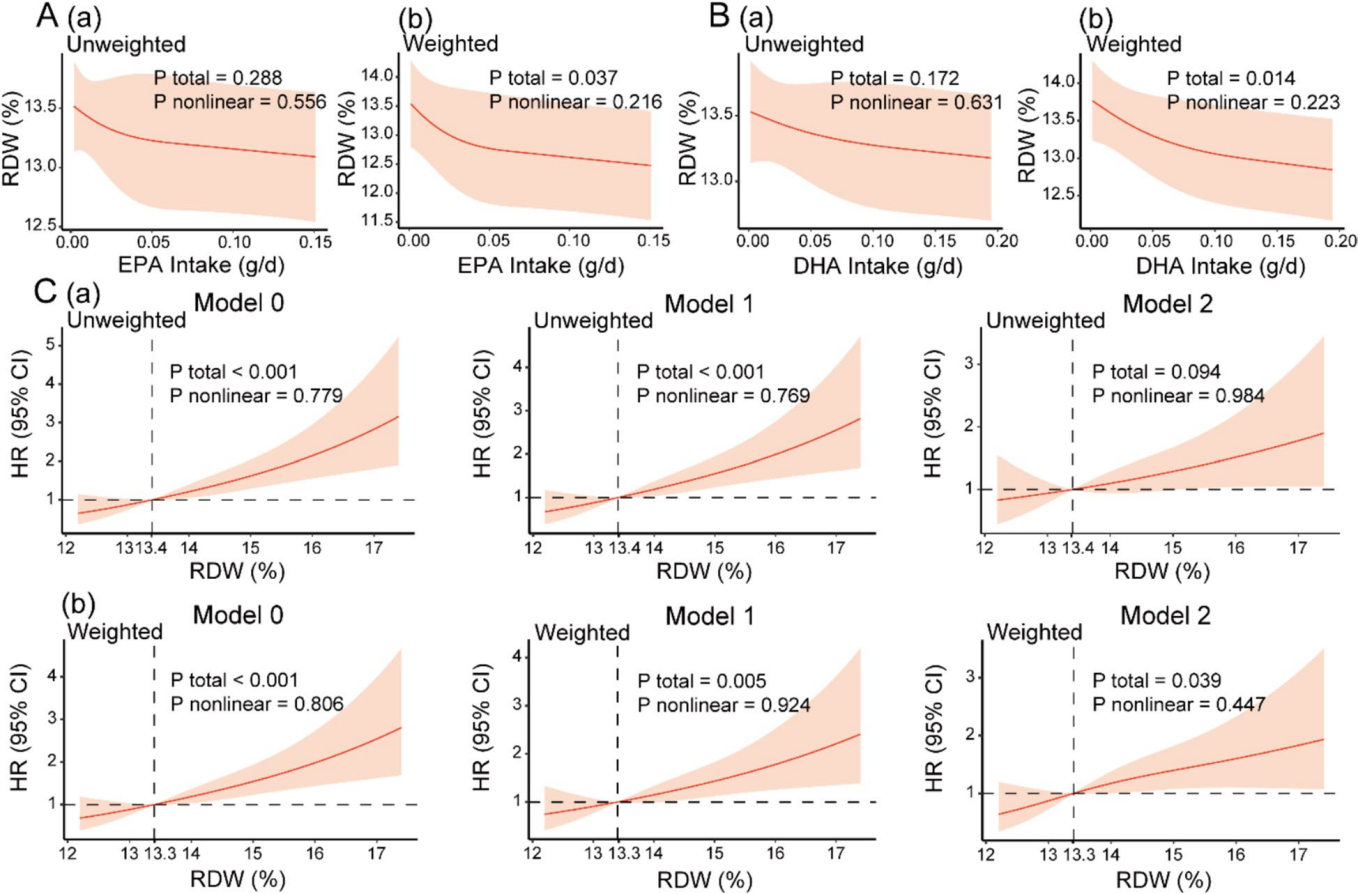
Associations between dietary n-3 FA intake and RDW, and between RDW and mortality. Panels labeled **a** show the unweighted analyses, and those labeled **b** show the weighted analyses. Model 0: unadjusted; Model 1: adjusted for age, sex, and race; Model 2: adjusted for all covariates. A and B. The RCSs based on linear regression show the associations between daily dietary intake of EPA and DHA and RDW. C. The RCSs based on Cox regression show the association between RDW and mortality. *RDW* red cell distribution width—coefficient of variation, *EPA* eicosapentaenoic acid, *DHA* docosahexaenoic acid, *HR* Hazard Ratio, *CI* confidence interval, *FA* fatty acid, *RCS* restricted cubic spline

Baseline characteristics are presented in Supplementary Tables S1-S3. RCS plots derived from linear regression models (Fig. 2A–B) showed that RDW gradually decreased with increasing dietary intake of EPA and DHA. These inverse trends remained statistically significant after applying survey-weighted analyses. No statistically significant nonlinear relationships were observed.

### RDW was positively associated with mortality

RCS plots derived from Cox models (Fig. 2C) revealed that mortality risk increased with higher RDW values. Statistically significant positive associations were observed in both Model 0 and Model 1, regardless of whether survey weights were applied. Model 2 also showed a statistically significant increasing trend after weighted analysis. No significant nonlinear relationships were detected.

### Higher dietary n-3 FA intake is associated with a trend toward lower mortality risk

The results of the Cox regression analysis examining the associations between dietary EPA and DHA intake and mortality are presented in Supplementary Table S4. Across all models, higher dietary n-3 FA intake was associated with a declining trend in mortality risk, although none of these associations reached statistical significance.

### Associations between n-3 blood biomarkers, RDW, and 5-year mortality following MI

#### N-3 blood biomarker levels are higher in the low-RDW group

Comparison of baseline characteristics between participants with high (≥ 13.4%) and low (< 13.4%) RDW (Table S5) revealed that the low-RDW group had higher levels of most n-3 blood biomarkers, lower 5-year mortality, and lower CRP concentrations.

### N-3 blood biomarkers were inversely associated with RDW

**Fig. 3:**
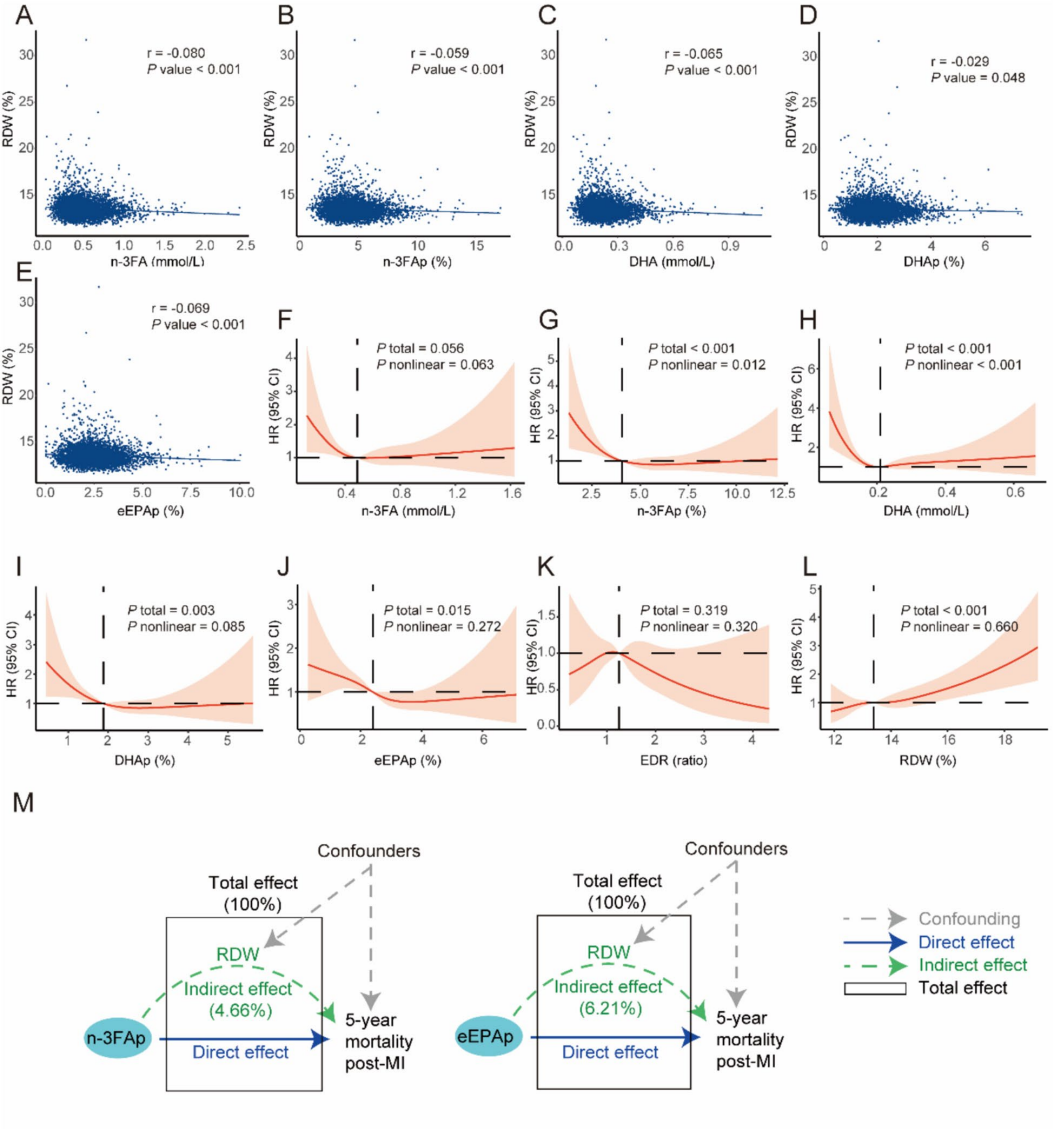
Associations between n-3 blood biomarkers, RDW, and 5 year mortality. A to E Show the correlations between RDW and n-3 blood biomarkers, which were confirmed by Spearman's correlation analysis. F to L Show the RCSs based on Cox regression for n-3 blood biomarkers and RDW in relation to the 5 year mortality. M shows the potential mediating role of RDW. RDW, red cell distribution width—coefficient of variation; n-3FA, omega-3 fatty acids; n-3FAp, omega-3 fatty acids to total fatty acids percentage; *DHA* docosahexaenoic acid; *DHAp* docosahexaenoic acid to total fatty acids percentage, *eEPAp* estimated eicosapentaenoic acid to total fatty acids percentage; *EDR* eEPAp/DHAp, *MI* myocardial infarction, *RCS* restricted cubic spline, *HR* hazard ratio, *CI* confidence interval

Scatter plots with fitted regression lines (Fig. 3A–E) illustrate negative associations between RDW and n-3 blood biomarkers. Spearman's correlation analysis confirmed these patterns, yielding statistically significant negative correlation coefficients (all *p* < 0.05). RCSs further revealed significant nonlinear inverse associations between RDW and n-3 blood biomarkers (Figure S1). Additionally, the change in RDW per SD increase in each standardized n-3 blood biomarker is detailed in Table S6.

### N-3 blood biomarkers were inversely associated, and RDW was positively associated, with 5-year mortality

RCSs based on Cox regression models (Fig. 3F–L) indicated that most n-3 blood biomarkers—including n-3FAp, DHA, DHAp, and eEPAp—were significantly inversely associated with 5-year mortality, with the associations for n-3FAp and DHA showing significant nonlinearity. In contrast, RDW exhibited a significant positive association with 5-year mortality, though this relationship did not display notable nonlinearity. The quantitative changes in mortality risk per SD increase in each n-3 biomarker or RDW are detailed in Table S7.

### RDW may partially mediate the associations between n-3 blood biomarkers and 5-year mortality

KHB mediation analysis indicated that RDW may partially mediate these associations, accounting for 4.66% of the effect between n-3FAp and 5 year mortality and 6.21% of the effect between eEPAp and 5 year mortality (Fig. 3M and Table 1).

**Table 1:** RDW in the associations between n-3 blood biomarkers and 5-year mortality (KHB mediation analysis)

Fatty acid biomarkers	Total effect		Direct effect		Indirect effect		Indirect effect/
	Coef (95% CI)	*P* value	Coef (95% CI)	*P* value	Coef (95% CI)	*P* value	Total effect (%)
n-3FA	− 0.850 (− 1.649, − 0.051)	0.037*	− 0.804 (− 1.607, 0.000)	0.050	− 0.046 (− 0.086, − 0.006)	0.036*	4.90
n-3FAp	− 0.124 (− 0.227, −0.021)	0.019*	− 0.118 (− 0.221, − 0.015)	0.025*	− 0.006 (− 0.011, − 0.001)	0.032*	4.66#
DHA	− 2.112 (− 4.009, − 0.215)	0.048*	− 1.879 (− 3.874, 0.116)	0.068	− 0.114 (− 0.215, − 0.013)	0.021*	5.40
DHAp	− 0.301 (− 0.550, − 0.052)	0.018*	− 0.293 (− 0.543, − 0.044)	0.021*	− 0.007 (− 0.017, 0.002)	0.123	2.54
eEPAp	− 0.180 (− 0.341, − 0.019)	0.028*	− 0.169 (− 0.330, − 0.007)	0.040*	− 0.011 (− 0.020, − 0.001)	0.022*	6.21#

*p < 0.05

#*P* values for the total effect, direct effect, and indirect effect are all less than 0.05.

RDW, red cell distribution width—coefficient of variation; n-3FA, omega-3 fatty acids; n-3FAp, omega-3 fatty acids to total fatty acids percentage; DHA, docosahexaenoic acid; DHAp, docosahexaenoic acid to total fatty acids percentage; eEPAp, estimated eicosapentaenoic acid to total fatty acids percentage; Coef, coefficient; CI, confidence interval.

### RDW may partially mediate the association between plasma EPA level and 6 month MACE following MI

In the internal dataset, KHB mediation analysis suggested a potential partial mediating role of RDW in the association between plasma EPA level and 6-month MACE incidence after MI, with the indirect effect accounting for 14.41% of the total effect (Table 2). Although the mediated effect did not reach statistical significance—likely due to limited statistical power from the small sample size—the indirect effect explained more than 10% of the total effect, suggesting potential clinical relevance despite the lack of definitive statistical evidence.

**Table 2:** RDW in the association between EPA and 6-month MACE incidence post-MI (KHB mediation analysis)

Total *n* = 55 events *n* = 10	Coefficient	Std. err	*z*	*P* value	95% CI		Indirect effect/Total effect (%)
					Low	High	
Total effect	− 1.779	1.048	− 1.70	0.089	− 3.833	0.274	
Direct effect	− 1.523	0.921	− 1.65	0.098	− 3.328	0.282	
Indirect effect	− 0.256	0.186	− 1.38	0.169	− 0.622	0.109	14.41

Without adjustment for covariates

*RDW* red cell distribution width—coefficient of variation, *EPA* eicosapentaenoic acid; *MACE* major adverse cardiovascular events, *MI* myocardial infarction, *CI* confidence interval

### Sensitivity analyses

We conducted sensitivity analyses by expanding the covariate sets in our multivariable models. In the NHANES cohort, we further adjusted for monthly family income (as a proxy for socioeconomic status) and a composite diet quality score (reflecting overall dietary patterns and calculated as previously described [29]) (Table S8). The direction of the associations remained virtually unchanged (Figure S2). Similarly, in the UK Biobank cohort, we evaluated progressively adjusted models incorporating increasingly comprehensive sets of potential confounders and consistently observed stable effect directions across all model specifications. Together, these analyses support the robustness of our findings.

Acknowledging that all approaches to handling missing data may introduce bias, we conducted a complete-case analysis in the UK Biobank cohort, restricting the sample to 3,230 participants with fully observed baseline covariates. The results remained consistent with those from the full cohort (Tables S6–S7, S9, S11; Figures S1G–L, S3), likely owing to the low prevalence and apparent randomness of missingness—suggestive of minimal selection bias—and the robustness of our statistical models. In contrast, a complete-case analysis was not feasible in the NHANES cohort due to an insufficient number of complete cases.

We further conducted sensitivity analyses of the mediation model in the UK Biobank cohort (Tables S10–S11). The indirect effect of RDW was notably stronger in models unadjusted for CRP but attenuated after adjustment for CRP. A consistent pattern was observed in the complete-case analysis. These findings suggest that CRP may partially mediate the association involving RDW, likely through shared inflammatory pathways, thereby providing supportive evidence for an anti-inflammatory mechanism underlying the cardioprotective effects of n-3 FAs.

## Discussion

### Association between RDW and prognosis following MI

RDW reflects the variability in erythrocyte volume. Previous research in a similar population has demonstrated a significant association between elevated RDW level and increased 5-year mortality risk among patients with prior MI [3]. Another study further reported that 6-month mortality is significantly higher in patients with MI who exhibit elevated RDW [30]. This association is not only linked to red blood cell dysfunction [5], but also reflects a systemic inflammatory state [31], which can promote myocardial fibrosis [32] and adverse left ventricular remodeling after MI [33, 34]. Specifically, sustained inflammation following MI may activate proteases and cardiac fibroblasts, upregulate cytokine expression, and enhance extracellular matrix deposition [35], resulting in expanded fibrotic areas, cardiomyocyte apoptosis, and increased ventricular stiffness—processes that collectively contribute to the development of heart failure. Notably, in the present study, participants in the high-RDW group within the UK Biobank cohort exhibited significantly higher CRP levels, further supporting the role of inflammation in adverse post-MI prognosis. Consistent with prior evidence, our findings reinforce RDW as a marker of poor prognosis. Given its ready availability and cost-effectiveness from EHRs, RDW represents an ideal tool for cardiovascular clinicians in monitoring and managing patients after MI.

### Associations between n-3 FAs and RDW

McBurney et al. demonstrated a negative correlation between blood n-3 status and RDW across all UK Biobank participants [36], but did not examine the associations of individual n-3 blood biomarkers—particularly DHA and EPA—with RDW. Similarly, Takahashi et al. [37] previously reported a significant reduction in RDW among 66 patients with ischemic heart disease following treatment with purified EPA. Despite these findings, few studies have specifically addressed these relationships in post-MI populations, leaving a notable gap in the literature. To address this gap, we conducted the present study within large post-MI cohorts. Our analyses confirmed inverse associations between dietary n-3 FA intake and RDW, as well as between circulating n-3 blood biomarkers and RDW. The availability of EHRs from large-scale databases provides extensive real-world health data, enabling researchers to detect subtle yet clinically relevant correlations among biomarkers.

### Cardioprotective effects of n-3 FAs

Our findings suggest that n-3 FAs may confer cardiovascular protection, although recent large-scale trials have yielded conflicting results [9–11]. Notably, unlike EPA, DHA has been hypothesized to lack cardiovascular benefits—or even to attenuate the protective effects of EPA [38]. Consistent with this hypothesis, our mediation analyses indicated that EPA-specific biomarker (eEPAp) accounted for a larger proportion of the association with clinical outcomes than DHA-related biomarkers, potentially explaining the inconsistent evidence across studies, which often differ in their EPA-to-DHA ratios. Although the EPA-to-DHA ratio (EDR)—a measure of their relative abundance—was not statistically significant in our analysis, it showed a consistent inverse association with mortality. This aligns with emerging evidence suggesting that the EPA:DHA balance, rather than absolute levels alone, may influence cardiovascular risk. Intriguingly, all participants exhibited low EDR values, indicating a population-level relative deficiency of EPA compared with DHA. Whether selectively increasing EDR through EPA-predominant supplementation confers additional clinical benefit remains an open question warranting focused investigation.

In our study, dietary intake of n-3 FAs was not significantly associated with mortality, whereas n-3 blood biomarkers demonstrated protective associations. This discrepancy likely stems from several factors. First, interindividual differences in absorption, bioavailability, and metabolism weaken the correlation between self-reported dietary intake and circulating biomarker levels. Second, dietary assessment in NHANES relies on 24-h recalls, which are susceptible to measurement error, day-to-day variation, and recall bias—limiting their capacity to reflect long-term n-3 FA exposure. Indeed, prior studies have reported only modest correlations (r ≈ 0.03–0.37) between self-reported fish or EPA/DHA intake and actual blood concentrations [39]. Furthermore, post-MI dietary changes—driven by clinical recommendations, lifestyle modifications, or altered appetite—are poorly captured in NHANES’ cross-sectional design. In contrast, blood n-3 levels integrate dietary intake over weeks to months and provide a more objective measure of exposure. Our findings underscore the limitations of dietary recall methods in nutrient–outcome research and highlight the superiority of biomarker-based approaches for evaluating the cardioprotective effects of n-3 FAs.

Here, we further underscore the objectivity of n-3 blood biomarkers from an alternative perspective: the NHANES cohort is inherently vulnerable to recall bias. Specifically, participants may underreport consumption of foods perceived as unhealthy—such as fried fish or processed sources—or overreport intake of foods deemed beneficial—such as fatty fish rich in EPA and DHA—a phenomenon known as “*social desirability bias*”. However, given the complexity of modern diets and limited public awareness of specific FA sources, underreporting generally predominates, particularly for episodically consumed items like fish. This non-differential misclassification would bias the observed association between self-reported n-3 FA intake and health outcomes toward the null, potentially explaining the null findings in our dietary analyses. In contrast, blood biomarkers are immune to such reporting biases and therefore offer a more objective assessment of exposure.

In summary, a growing consensus holds that n-3 FAs—particularly EPA—exert significant cardioprotective effects through multiple pleiotropic mechanisms [8, 40–42]. These FAs are thought to promote uniform cholesterol distribution within cell membranes and reduce peroxyl radical mobility, thereby stabilizing endothelial and immune cells; decrease the formation of cholesterol crystalline domains, contributing to the stabilization—and potentially even the regression—of atherosclerotic plaques; inhibit platelet aggregation; and compete with arachidonic acid to confer anti-inflammatory effects that mitigate systemic inflammation. Further research is warranted to fully elucidate these complex mechanisms.

### Potential mediating role of RDW

Our results show an inverse association between n-3 FAs and RDW in post-MI patients, consistent with the notion that n-3 FAs may attenuate inflammation and improve erythrocyte function. Exploratory mediation analyses in both the UK Biobank and our internal cohort suggest a modest mediating role of RDW in the association between n-3 FAs—particularly EPA—and cardiovascular outcomes.

However, RDW likely functions as a biomarker of underlying inflammation and metabolic disturbances rather than as a direct causal factor. The observed mediation effect may reflect the extent to which EPA ameliorates these pathological states, thereby lowering RDW. Consequently, a reduction in RDW may signify improved inflammatory and erythrocyte status rather than representing a therapeutic mechanism per se. Our findings support an associative, biologically plausible pathway—but not causality. Future interventional studies are needed to determine whether the cardioprotective effects of n-3 FAs operate through RDW-related mechanisms.

### Strengths and limitations

The key strength is the use of large public EHR databases, enabling a large sample size and long follow-up to detect subtle, long-term associations. Nevertheless, several limitations warrant consideration. First, as an observational study, it lacks control over participants’ n-3 FA intake, and despite extensive statistical adjustment, residual confounding cannot be excluded. Second, the temporal sequence among MI onset, biomarker assessment, and outcome ascertainment is unclear in the public cohorts: NHANES relies on self-reported MI without timing information, while UK Biobank’s biomarker measurements may not capture the acute or early post-MI physiological state. Third, excluding UK Biobank participants with < 5 years of follow-up may introduce selection bias if excluded individuals differ systematically from those retained in baseline health or socioeconomic characteristics. Fourth, reliance on questionnaire-derived variables may introduce recall bias, and measurement error in dietary data likely attenuated associations, reducing statistical power to detect true effects. Fifth, the KHB method required dichotomizing 5 year mortality, discarding time-to-event information and potentially reducing statistical efficiency and obscuring temporal dynamics. While this simplification enabled exploratory mediation analysis, future work will apply modern survival mediation approaches that preserve the time-to-event structure. Sixth, the mediation analysis for 6-month MACE in the internal cohort was unadjusted for potential confounders, rendering it prone to residual confounding; thus, findings should be considered exploratory and require validation in larger studies. Seventh, while a partial mediating role of RDW was observed, the underlying biological mechanisms were not investigated—yet elucidating these pathways is essential to substantiate the proposed causal framework.

## Conclusions

In individuals with prior MI, both dietary intake and blood levels of n-3 FAs were inversely associated with RDW. Higher n-3 blood biomarkers were also associated with lower mortality risk. Building on these findings, mediation analyses suggested that RDW may partially account for the observed association between blood n-3 FAs—particularly EPA—and improved prognosis after MI. These findings align with hypothesized biological pathways—such as anti-inflammatory effects and improved erythrocyte function—through which n-3 FAs might influence post-MI outcomes. However, given the observational nature of this study, causal inference remains tentative and requires validation in randomized controlled trials.

## Supplementary Information

Supplementary material 1. Table S1. Baseline characteristics of the NHANES cohort. Table S2. Baseline characteristics by RDW group in the NHANES cohort. Table S3. Baseline characteristics by RDW group in the NHANES cohort. Table S4. Cox models of dietary n-3 FAs, RDW and mortality in the NHANES cohort. Table S5. Baseline characteristics by RDW group in the UK Biobank cohort. Table S6. Associations between RDW and n-3 blood biomarkers in the UK Biobank cohort. Table S7. Cox models of n-3 biomarkers, RDW and 5-year mortality in the UK Biobank cohort. Table S8. Baseline characteristics of additional covariates in the NHANES cohort. Table S9. RDW in the associations between n-3 blood biomarkers and 5-year mortality post-MI. Table S10. RDW in the associations between n-3 blood biomarkers and 5-year mortality post-MI. Table S11. RDW in the associations between n-3 blood biomarkers and 5-year mortality post-MI. Figure S1. Associations between n-3 blood biomarkers and RDW: RCSs based on linear regression. Figure S2. Adjusted associations between dietary n-3 FAs, RDW, and mortality. Figure S3. RCSs from Cox models of n-3 biomarkers and RDW on 5-year mortality

## Data Availability

NHANES: [https://wwwn.cdc.gov/nchs/nhanes/default.aspx]; NDI Mortality Files: [https://www.cdc.gov/nchs/data-linkage/mortality-public.htm]; UK Biobank: [http://www.ukbiobank.ac.uk]; Internal dataset may be available upon reasonable request from the corresponding author via email.

## Abbreviations

EHRs: Electronic health records
RDW: Red cell distribution width—coefficient of variation
MI: Myocardial infarction
n-3 FAs: Omega-3 fatty acids
MACE: Major adverse cardiovascular events
NHANES: The National Health and Nutrition Examination Survey
EPA: Eicosapentaenoic acid
IPE: Icosapent ethyl
DHA: Docosahexaenoic acid
PIR: Ratio of family income to poverty
BMI: Body mass index
TG: Triglycerides
HDL-C: High-density lipoprotein cholesterol
LDL-C: Low-density lipoprotein cholesterol
SBP: Systolic blood pressure
DBP: Diastolic blood pressure
CRP: C-reactive protein
ACEIs/ARBs: Angiotensin-converting enzyme inhibitors and angiotensin II receptor blockers
CCBs: Calcium channel blockers
n-3FA: Omega-3 fatty acids
n-3FAp: Omega-3 fatty acids to total fatty acids percentage
DHAp: Docosahexaenoic acid to total fatty acids percentage
eEPAp: Estimated eicosapentaenoic acid to total fatty acids percentage
EDR: EEPAp/DHAp
RCS: Restricted cubic spline
KHB: Karlson–Holm–Breen
SD: Standard deviation
HR: Hazard ratio
CI: Confidence interval
Coef: Coefficient
