# Supplementary material for: Association of omega-3 fatty acids with improved prognosis after myocardial infarction: the role of red cell distribution width—an EHR study
Source: Eur J Med Res. 2026 Jan 21;31:303. doi: 10.1186/s40001-026-03909-9 (PMC12908392; doi:10.1186/s40001-026-03909-9)
Supplement: Supplementary file 1 — Supplementary material 1. Table S1. Baseline characteristics of the NHANES cohort. Table S2. Baseline characteristics by RDW group in the NHANES cohort. Table S3. Baseline characteristics by RDW group in the NHANES cohort. Table S4. Cox models of dietary n-3 FAs, RDW and mortality in the NHANES cohort. Table S5. Baseline characteristics by RDW group in the UK Biobank cohort. Table S6. Associations between RDW and n-3 blood biomarkers in the UK Biobank cohort. Table S7. Cox models of n-3 biomarkers, RDW and 5-year mortality in the UK Biobank cohort. Table S8. Baseline characteristics of additional covariates in the NHANES cohort. Table S9. RDW in the associations between n-3 blood biomarkers and 5-year mortality post-MI. Table S10. RDW in the associations between n-3 blood biomarkers and 5-year mortality post-MI. Table S11. RDW in the associations between n-3 blood biomarkers and 5-year mortality post-MI. Figure S1. Associations between n-3 blood biomarkers and RDW: RCSs based on linear regression. Figure S2. Adjusted associations between dietary n-3 FAs, RDW, and mortality. Figure S3. RCSs from Cox models of n-3 biomarkers and RDW on 5-year mortality [file 40001_2026_3909_MOESM1_ESM.docx]

List of Supplementary Tables and Figure Legends

Table S1. Baseline characteristics of the NHANES cohort.

| Characteristics | All Participants | |
| --- | --- | --- |
|  | Unweighted (n=230) | Weighted^a^ (n=857776) |
| **Demographics Data** |  |  |
| Age (years) | 68 (60, 76) | 65 (57, 74) |
| Gender (male, n [%]) | 164 [71.30%] | 533279 [62.17%] |
| Race |  |  |
| Mexican American (n [%]) | 19 [8.30%] | 17670 [2.06%] |
| Other Hispanic (n [%]) | 25 [10.85%] | 47864 [5.58%] |
| Non-Hispanic White (n [%]) | 117 [50.85%] | 585689 [68.28%] |
| Non-Hispanic Black (n [%]) | 46 [20.00%] | 96586 [11.26%] |
| Other Race (n [%]) | 23 [10.00%] | 109967 [12.82%] |
| PIR (ratio) | 1.92 (1.09, 2.91) | 2.12 (1.39, 2.75) |
| **Examination Data** |  |  |
| BMI (kg/m^2^) | 28.5 (25.3, 32.1) | 27.8 (24.8, 31.1) |
| **Laboratory Data** |  |  |
| RDW (%) | 13.4 (13.0, 14.2) | 13.3 (12.8, 13.9) |
| HDL-C (mmol/L) | 1.19 (1.03, 1.50) | 1.24 (1.06, 1.60) |
| Total cholesterol (mmol/L) | 4.19 (3.62, 5.09) | 4.65 (3.72, 5.35) |
| TG (mmol/L) | 1.214 (0.903, 1.671) | 1.197 (0.881, 1.648) |
| LDL-C (mmol/L) | 2.302 (1.790, 2.994) | 2.483 (1.862, 3.284) |
| **Questionnaire Data** |  |  |
| Alcohol (Yes, n [%]) | 158 [68.70%] | 612281 [71.38%] |
| Smoker (Yes, n [%]) | 147 [63.91%] | 580371 [67.66%] |
| **Comorbidities** |  |  |
| Hypertension (Yes, n [%]) | 163 [70.87%] | 599242 [69.86%] |
| Diabetes (Yes, n [%]) | 78 [33.91%] | 214787 [25.04%] |
| Kidney Disease (Yes, n [%]) | 18 [7.83%] | 80288 [9.36%] |
| Congestive Heart Failure (Yes, n [%]) | 65 [28.26%] | 215988 [25.18%] |
| Stroke (Yes, n [%]) | 38 [16.52%] | 163321 [19.04%] |
| **Nutrient Intakes** |  |  |
| Total Energy (kcal/d) | 1794 (1290, 2263) | 1838 (1432, 2364.5) |
| EPA (20:5) (g/d) | 0.007 (0.003, 0.015) | 0.007 (0.003, 0.014) |
| DHA (22:6) (g/d) | 0.023 (0.006, 0.051) | 0.021 (0.004, 0.051) |

a. Weighted data excluded the sampled population corresponding to strata that contain only a single Primary Sampling Unit (PSU).

PIR, ratio of family income to poverty; BMI, body mass index; RDW, red cell distribution width - coefficient of variation; HDL-C, high-density lipoprotein cholesterol; TG, triglycerides; LDL-C, low-density lipoproteins cholesterol; EPA, eicosapentaenoic acid; DHA, docosahexaenoic acid.

Table S2. Baseline characteristics by RDW group in the NHANES cohort.

| Characteristics | RDW (<13.4%) (n=103) | RDW (≥13.4%) (n=127) | *P* value |  |
| --- | --- | --- | --- | --- |
| **Demographics Data** |  |  |  |  |
| Age (years) | 66 (59, 75) | 69 (61, 77) | 0.161 |  |
| Gender (male, n [%]) | 74 [71.8%] | 90 [70.9%] | 0.987 |  |
| Race |  |  | <0.001 | *** |
| Mexican American (n [%]) | 8 [7.8%] | 11 [8.7%] |  |  |
| Other Hispanic (n [%]) | 13 [12.6%] | 12 [9.5%] |  |  |
| Non-Hispanic White (n [%]) | 55 [53.4%] | 62 [48.8%] |  |  |
| Non-Hispanic Black (n [%]) | 12 [11.7%] | 34 [26.8%] |  |  |
| Other Race (n [%]) | 15 [14.6%] | 8 [6.3%] |  |  |
| PIR (ratio) | 1.96 (1.32, 2.75) | 1.79 (0.99, 2.99) | 0.230 |  |
| **Examination Data** |  |  |  |  |
| BMI (kg/m^2^) | 28.8 (25.75, 31.34) | 28.3 (25.00, 32.38) | 0.785 |  |
| **Laboratory Data** |  |  |  |  |
| RDW (%) | 12.8 (12.5, 13.1) | 14.0 (13.7, 14.9) | <0.001 | *** |
| HDL-C (mmol/L) | 1.22 (1.03, 1.53) | 1.16 (1.02, 1.46) | 0.192 |  |
| Total Cholesterol (mmol/L) | 4.24 (3.70, 5.12) | 4.14 (3.47, 4.94) | 0.106 |  |
| TG (mmol/L) | 1.33 (1.03, 1.67) | 1.14 (0.85, 1.68) | 0.025 | * |
| LDL-C (mmol/L) | 2.30 (1.86, 2.94) | 2.30 (1.71, 3.01) | 0.685 |  |
| **Questionnaire Data** |  |  |  |  |
| Alcohol (Yes, n [%]) | 74 [71.8%] | 84 [66.1%] | <0.001 | *** |
| Smoker (Yes, n [%]) | 62 [60.2%] | 85 [66.9%] | 0.358 |  |
| **Comorbidities** |  |  |  |  |
| Hypertension (Yes, n [%]) | 71 [68.9%] | 92 [72.4%] | <0.001 | *** |
| Diabetes (Yes, n [%]) | 23 [22.3%] | 55 [43.3%] | <0.001 | *** |
| Kidney Disease (Yes, n [%]) | 6 [5.8%] | 12 [9.5%] | <0.001 | *** |
| Heart Failure (Yes, n [%]) | 21 [20.4%] | 44 [34.6%] | <0.001 | *** |
| Stroke (Yes, n [%]) | 13 [12.6%] | 25 [19.7%] | <0.001 | *** |
| **Nutrient Intakes** |  |  |  |  |
| Total energy (kcal/d) | 1830.5 (1438.5, 2145.8) | 1688.0 (1220.0, 2295.3) | 0.291 |  |
| EPA (20:5) (g/d) | 0.007 (0.003, 0.014) | 0.008 (0.004, 0.015) | 0.441 |  |
| DHA (22:6) (g/d) | 0.027 (0.006, 0.052) | 0.022 (0.006, 0.051) | 0.911 |  |

*** *p* < 0.001; * *p* < 0.05.

PIR, ratio of family income to poverty; BMI, body mass index; RDW, red cell distribution width - coefficient of variation; HDL-C, high-density lipoprotein cholesterol; TG, triglycerides; LDL-C, low-density lipoproteins cholesterol; EPA, eicosapentaenoic acid; DHA, docosahexaenoic acid.

Table S3. Baseline characteristics by RDW group in the NHANES cohort (weighted).

| Characteristics | RDW (<13.4%) (n=425318) | RDW (≥13.4%) (n=432458) | *P* value |  |
| --- | --- | --- | --- | --- |
| **Demographics Data** |  |  |  |  |
| Age (years) | 62 (55, 71) | 67 (59, 76) | <0.001 | *** |
| Gender (male, n [%]) | 265950 [62.53%] | 267329 [61.82%] | <0.001 | *** |
| **Race** |  |  | <0.001 | *** |
| Mexican American (n [%]) | 10648 [2.50%] | 7022 [1.62%] |  |  |
| Other Hispanic (n [%]) | 26820 [6.30%] | 21044 [4.86%] |  |  |
| Non-Hispanic White (n [%]) | 302980 [71.23%] | 282709 [65.36%] |  |  |
| Non-Hispanic Black (n [%]) | 41038 [9.64%] | 55548 [12.84%] |  |  |
| Other Race (n [%]) | 43817 [10.30%] | 66150 [15.30%] |  |  |
| PIR (ratio) | 2.26 (1.54, 2.83) | 1.99 (1.25, 2.67) | <0.001 | *** |
| **Examination Data** |  |  |  |  |
| BMI (kg/m²) | 27.4 (24.6, 30.7) | 28.2 (25.0, 31.5) | <0.001 | *** |
| **Laboratory Data** |  |  |  |  |
| RDW (%) | 12.6 (12.3, 12.9) | 13.9 (13.5, 14.7) | <0.001 | *** |
| HDL-C (mmol/L) | 1.29 (1.11, 1.66) | 1.19 (1.01, 1.54) | <0.001 | *** |
| Total Cholesterol (mmol/L) | 4.74 (3.81, 5.43) | 4.56 (3.63, 5.27) | <0.001 | *** |
| Triglycerides (mmol/L) | 1.26 (0.93, 1.71) | 1.14 (0.83, 1.59) | <0.001 | *** |
| LDL-C (mmol/L) | 2.53 (1.93, 3.36) | 2.44 (1.81, 3.22) | 0.002 | ** |
| **Questionnaire Data** |  |  |  |  |
| Alcohol (Yes, n [%]) | 319009 [74.99%] | 293272 [67.81%] | <0.001 | *** |
| Smoke (Yes, n [%]) | 278455 [65.47%] | 301916 [69.81%] | <0.001 | *** |
| **Comorbidities** |  |  |  |  |
| Hypertension (Yes, n [%]) | 286341 [67.32%] | 312901 [72.35%] | <0.001 | *** |
| Diabetes (Yes, n [%]) | 92338 [21.71%] | 122449 [28.31%] | <0.001 | *** |
| Kidney Disease (Yes, n [%]) | 34045 [8.00%] | 46243 [10.69%] | <0.001 | *** |
| Congestive Heart Failure (Yes, n [%]) | 96338 [22.65%] | 119650 [27.66%] | <0.001 | *** |
| Stroke (Yes, n [%]) | 72324 [17.00%] | 90997 [21.03%] | <0.001 | *** |
| **Nutrient Intakes** |  |  |  |  |
| Total Energy (kcal/d) | 1895 (1495, 2420) | 1782 (1371, 2310) | <0.001 | *** |
| EPA (20:5) (g/d) | 0.008 (0.004, 0.016) | 0.006 (0.002, 0.012) | <0.001 | *** |
| DHA (22:6) (g/d) | 0.025 (0.006, 0.056) | 0.017 (0.003, 0.046) | <0.001 | *** |

*** *p* < 0.001; ** *p* < 0.01.

PIR, ratio of family income to poverty; BMI, body mass index; RDW, red cell distribution width - coefficient of variation; HDL-C, high-density lipoprotein cholesterol; TG, triglycerides; LDL-C, low-density lipoproteins cholesterol; EPA, eicosapentaenoic acid; DHA, docosahexaenoic acid.

Table S4. Cox models of dietary n-3 FAs, RDW and mortality in the NHANES cohort (per SD).

|  | Model 0 | |  | Model 1 | |  | Model 2 | |
| --- | --- | --- | --- | --- | --- | --- | --- | --- |
| Factors | HR (95%CI) | *P* value |  | HR (95%CI) | *P* value |  | HR (95%CI) | *P* value |
| **Unweighted** |  |  |  |  |  |  |  |  |
| RDW | 1.190 (1.030-1.375) | 0.018 | * | 1.390 (1.143-1.692) | <0.001 | *** | 1.318 (1.051-1.652) | 0.016 * |
| EPA | 0.920 (0.723-1.170) | 0.497 |  | 0.928 (0.710-1.836) | 0.896 |  | 0.976 (0.712-1.421) | 0.695 |
| DHA | 0.907 (0.711-1.158) | 0.435 |  | 0.950 (0.722-1.249) | 0.714 |  | 0.971 (0.744-1.266) | 0.829 |
| dEDR | 0.815 (0.625-1.168) | 0.224 |  | 0.892 (0.680-1.314) | 0.357 |  | 0.910 (0.723-1.223) | 0.487 |
| **Weighted** |  |  |  |  |  |  |  |  |
| RDW | 1.258 (1.140–1.455) | <0.001 | *** | 1.120 (1.050–1.470) | <0.001 | *** | 1.362 (1.165–1.490) | 0.009** |
| EPA | 0.440 (0.070–1.400) | 0.660 |  | 0.885 (0.130–1.360) | 0.750 |  | 0.615 (0.022–1.550) | 0.665 |
| DHA | 0.630 (0.125–1.430) | 0.330 |  | 0.685 (0.057–1.360) | 0.570 |  | 0.590 (0.185–1.450) | 0.464 |
| dEDR | 0.850 (0.325–1.060) | 0.135 |  | 0.840 (0.555–1.245) | 0.230 |  | 0.895 (0.525–1.240) | 0.280 |

*** *p* < 0.001; ** *p* < 0.01; * *p* < 0.05.

Model 0: unadjusted; Model 1: adjusted for age, gender, and race; Model 2: adjusted for all covariables, and stratified according to the LDL-C (2.6 mmol/L), total cholesterol levels (5.2 mmol/L), and alcohol consumption status. All models satisfied the proportional hazards assumption.

FA, fatty acid; RDW, red cell distribution width - coefficient of variation; SD, standard deviation; EPA, dietary eicosapentaenoic acid; DHA, dietary docosahexaenoic acid; dEDR, dietary eicosapentaenoic acid / docosahexaenoic acid; HR, Hazard Ratio; CI, confidence interval.

Table S5. Baseline characteristics by RDW group in the UK Biobank cohort.

| Characteristics | RDW (<13.4%) (n=1944) | RDW (≥13.4%) (n=2099) | *P* value |  |
| --- | --- | --- | --- | --- |
| **Demographics** |  |  |  |  |
| Age (years) | 60 (54, 64) | 61 (56, 65) | <0.001 | *** |
| Sex (male, n [%]) | 1511 [77.7%] | 1613 [76.8%] | <0.001 | *** |
| TDI | -2.27 (-3.70, 0.28) | -1.98 (-3.49, 0.94) | 0.002 | ** |
| **Physical Examination** |  |  |  |  |
| BMI (kg/m^2^) | 27.60 (25.35, 30.38) | 28.20 (25.68, 31.38) | <0.001 | *** |
| SBP (mmHg) | 141 (129, 154) | 142 (129, 155) | 0.762 |  |
| DBP (mmHg) | 83 (75, 89) | 81 (74, 88) | 0.031 | * |
| **Lifestyle** |  |  |  |  |
| Smoking (Yes, n [%]) | 245 [12.6%] | 386 [18.4%] | <0.001 | *** |
| Diet score | 3.00 (2.00, 4.00) | 3.00 (2.00, 4.00) | 0.994 |  |
| **Laboratory Data** |  |  |  |  |
| RDW (%) | 13.0 (12.7, 13.2) | 13.9 (13.6, 14.4) | <0.001 | *** |
| CRP (mg/L) | 1.30 (0.69, 2.48) | 1.59 (0.81, 3.20) | <0.001 | *** |
| Total cholesterol (mmol/L) | 5.14 (4.25, 6.13) | 5.10 (4.14, 6.08) | 0.285 |  |
| Creatinine (umol/L) | 78.0 (69.0, 87.7) | 79.3 (69.5, 88.8) | 0.138 |  |
| Glucose (mmol/L) | 5.01 (4.65, 5.41) | 5.05 (4.71, 5.47) | 0.186 |  |
| HDL_C (mmol/L) | 1.19 (1.03, 1.38) | 1.17 (1.01, 1.39) | 0.542 |  |
| TG (mmol/L) | 1.82 (1.26, 2.58) | 1.73 (1.21, 2.42) | 0.009 | ** |
| **Comorbidities** |  |  |  |  |
| Atrial fibrillation (Yes, n [%]) | 277 [14.2%] | 384 [18.3%] | <0.001 | *** |
| Arrest (Yes, n [%]) | 29 [1.5%] | 35 [1.7%] | 0.748 |  |
| Arrhythmia (Yes, n [%]) | 544 [28.0%] | 650 [31.0%] | <0.001 | *** |
| Hypertension (Yes, n [%]) | 1298 [66.8%] | 1471 [70.1%] | <0.001 | *** |
| Heart failure (Yes, n [%]) | 63 [3.2%] | 94 [4.5%] | <0.001 | *** |
| Valvular disease (Yes, n [%]) | 303 [15.6%] | 353 [16.8%] | <0.001 | *** |
| **Medication Data** |  |  |  |  |
| Metformin (Yes, n [%]) | 103 [5.3%] | 163 [7.8%] | <0.001 | *** |
| Aspirin (Yes, n [%]) | 933 [48.0%] | 1103 [52.5%] | <0.001 | *** |
| Clopidogrel (Yes, n [%]) | 149 [7.7%] | 166 [7.9%] | <0.001 | *** |
| Statins (Yes, n [%]) | 896 [46.1%] | 1050 [50.0%] | <0.001 | *** |
| β-blocker (Yes, n [%]) | 353 [18.2%] | 387 [18.4%] | <0.001 | *** |
| ACEIs/ARBs (Yes, n [%]) | 802 [41.2%] | 939 [44.8%] | <0.001 | *** |
| CCBs (Yes, n [%]) | 203 [10.4%] | 276 [13.1%] | <0.001 | *** |
| **N-3 blood biomarkers** | |  |  |  |
| n-3FA (mmol/L) | 0.499 (0.394, 0.635) | 0.478 (0.365, 0.617) | <0.001 | *** |
| n-3FAp (%) | 4.34 (3.46, 5.32) | 4.23 (3.42, 5.20) | 0.033 | * |
| DHA (mmol/L) | 0.215 (0.172, 0.263) | 0.208 (0.166, 0.260) | 0.013 | * |
| DHAp (%) | 1.88 (1.49, 2.34) | 1.88 (1.46, 2.33) | 0.751 |  |
| eEPAp (%) | 2.46 (1.94, 3.12) | 2.37 (1.84, 3.02) | 0.002 | ** |
| EDR (ratio) | 1.30 (1.05, 1.60) | 1.27 (1.02, 1.57) | 0.013 | * |
| **Outcomes** |  |  |  |  |
| 5-year death (Yes, n [%]) | 98 [5.0%] | 137 [6.5%] | <0.001 | *** |

*** *p* < 0.001; ** *p* < 0.01; * *p* < 0.05.

RDW groups were defined using the cohort median (13.4%) as the cut-off.

RDW, red cell distribution width - coefficient of variation; TDI, Townsend deprivation index; BMI, body mass index; SBP, systolic blood pressure; DBP, diastolic blood pressure; CRP, C-reactive protein; HDL-C, high-density lipoprotein cholesterol; TG, triglycerides; ACEIs/ARBs, angiotensin-converting enzyme inhibitors and angiotensin II receptor blockers; CCBs, calcium channel blockers; n-3FA, omega-3 fatty acids; n-3FAp, omega-3 fatty acids to total fatty acids percentage; DHA, docosahexaenoic acid; DHAp, docosahexaenoic acid to total fatty acids percentage; eEPAp, estimated eicosapentaenoic acid to total fatty acids percentage; EDR, eEPAp/DHAp.

Table S6. Associations between RDW and n-3 blood biomarkers in the UK Biobank cohort (per SD).

|  | Original cohort (n=4043) | |  | Complete-case cohort (n=3230) | |  |
| --- | --- | --- | --- | --- | --- | --- |
| Factors | β (95% CI) | *P* value |  | β (95% CI) | *P* value |  |
| n-3FA | -0.074 (-0.107–-0.041) | <0.001 | *** | -0.078 (-0.115–-0.042) | <0.001 | *** |
| n-3FAp | -0.062 (-0.094–-0.031) | <0.001 | *** | -0.065 (-0.100–-0.030) | <0.001 | *** |
| DHA | -0.062 (-0.094–-0.028) | <0.001 | *** | -0.066 (-0.102–-0.029) | <0.001 | *** |
| DHAp | -0.042 (-0.078–-0.006) | 0.021 | * | -0.044 (-0.084–-0.005) | 0.026 | * |
| eEPAp | -0.074 (-0.105–-0.042) | <0.001 | *** | -0.077 (-0.112–-0.042) | <0.001 | *** |
| EDR | -0.066 (-0.112–-0.020) | 0.005 | ** | -0.063 (-0.114–-0.013) | 0.014 | * |

*** *p* < 0.001; ** *p* < 0.01; * *p* < 0.05.

β coefficients represent the difference in RDW (in original units - %) per SD higher biomarker level.

Models were adjusted for all covariables in the UK Biobank cohort, and stratified by creatinine levels (100 μmol/L), glucose levels (11 mmol/L), atrial fibrillation, arrest, and statin use. All models met the proportional hazards assumption.

RDW, red cell distribution width - coefficient of variation; SD, standard deviation; n-3FA, omega-3 fatty acids; n-3FAp, omega-3 fatty acids to total fatty acids percentage; DHA, docosahexaenoic acid; DHAp, docosahexaenoic acid to total fatty acids percentage; eEPAp, estimated eicosapentaenoic acid to total fatty acids percentage; EDR, eEPAp/DHAp; CI, confidence interval.

Table S7. Cox models of n-3 biomarkers, RDW and 5-year mortality in the UK Biobank cohort (per SD).

|  | Original cohort (n=4043) | |  | Complete-case cohort (n=3230) | |  |
| --- | --- | --- | --- | --- | --- | --- |
| Factors | HR (95%CI) | *P* value |  | HR (95%CI) | *P* value |  |
| RDW | 1.144 (1.042-1.255) | 0.004 | ** | 1.161 (1.031-1.307) | 0.013 | * |
| n-3FA | 0.871 (0.751-1.010) | 0.068 |  | 0.857 (0.720-1.021) | 0.084 |  |
| n-3FAp | 0.864 (0.750-0.994) | 0.041 | * | 0.836 (0.709-0.986) | 0.033 | * |
| DHA | 0.879 (0.763-1.014) | 0.075 |  | 0.833 (0.704-0.985) | 0.034 | * |
| DHAp | 0.852 (0.730-0.993) | 0.040 | * | 0.796 (0.661-0.959) | 0.016 | * |
| eEPAp | 0.861 (0.747-0.993) | 0.041 | * | 0.842 (0.714-0.992) | 0.040 | * |
| EDR | 0.877 (0.710-1.083) | 0.224 |  | 0.933 (0.722-1.205) | 0.598 |  |

** *p* < 0.01; * *p* < 0.05.

Models were adjusted for all covariables in the UK Biobank cohort, and stratified by creatinine levels (100 μmol/L), glucose levels (11 mmol/L), atrial fibrillation, arrest, and statin use. All models met the proportional hazards assumption.

RDW, red cell distribution width - coefficient of variation; SD, standard deviation; n-3FA, omega-3 fatty acids; n-3FAp, omega-3 fatty acids to total fatty acids percentage; DHA, docosahexaenoic acid; DHAp, docosahexaenoic acid to total fatty acids percentage; eEPAp, estimated eicosapentaenoic acid to total fatty acids percentage; EDR, eEPAp/DHAp; HR, Hazard Ratio; CI, confidence interval.

Table S8. Baseline characteristics of additional covariates in the NHANES cohort.

| Characteristics | All Participants | RDW (<13.4%) | RDW (≥13.4%) | *P* value |  |
| --- | --- | --- | --- | --- | --- |
| **Unweighted** | (n=230) | (n=103) | (n=127) |  |  |
| Monthly family income | 6.00 (4.00–7.00) | 6.00 (4.75–6.25) | 6.00 (4.00–8.00) | 0.599 |  |
| Diet score | 2.00 (1.50–3.00) | 2.25 (1.50-3.75) | 2.00 (1.00-4.00) | 0.683 |  |
| **Weighted** | (n=857776) | (n=425318) | (n=432458) |  |  |
| Monthly family income | 6.25 (4.75–7.25) | 6.50 (5.00–7.50) | 6.00 (4.50–7.00) | 0.017 | * |
| Diet score | 2 (1–3) | 2 (1–3) | 2 (1–2) | 0.248 |  |

* *p* < 0.05.

RDW, red cell distribution width - coefficient of variation.

Table S9. RDW in the associations between n-3 blood biomarkers and 5-year mortality post-MI (KHB mediation analysis, complete-case cohort, n = 3230).

| Fatty Acid | Total Effect | |  | Direct Effect | |  | | Indirect Effect | | Indirect Effect/ |
| --- | --- | --- | --- | --- | --- | --- | --- | --- | --- | --- |
| biomarkers | Coef (95% CI) | *P* value | Coef (95% CI) | | *P* value | | Coef (95% CI) | | *P* value | Total Effect (%) |
| n-3FA | -0.915 (-1.876, 0.045) | 0.062 |  | -0.871 (-1.832, 0.088) | 0.075 |  | | -0.043 (-0.092, 0.005) | 0.080 | 4.74 |
| n-3FAp | -0.137 (-0.261, -0.015) | 0.028* |  | -0.132 (-0.254, -0.009) | 0.035* |  | | -0.005 (-0.012, 0.001) | 0.070 | 4.25 |
| DHA | -2.136 (-4.278, 0.006) | 0.052 |  | -1.830 (-3.724, 0.064) | 0.058 |  | | -0.104 (-0.198, -0.010) | 0.023* | 4.87 |
| DHAp | -0.306 (-0.603, -0.011) | 0.042* |  | -0.300 (-0.596, -0.004) | 0.047* |  | | -0.006 (-0.019, 0.005) | 0.297 | 2.17 |
| eEPAp | -0.214 (-0.408, -0.021) | 0.030* |  | -0.203 (-0.396, -0.009) | 0.040* |  | | -0.012 (-0.023, -0.001) | 0.043* | 5.53# |

* *p* < 0.05.

# *P* values for the total effect, direct effect, and indirect effect are all less than 0.05.

RDW, red cell distribution width - coefficient of variation; MI, myocardial infarction; n-3FA, omega-3 fatty acids; n-3FAp, omega-3 fatty acids to total fatty acids percentage; DHA, docosahexaenoic acid; DHAp, docosahexaenoic acid to total fatty acids percentage; eEPAp, estimated eicosapentaenoic acid to total fatty acids percentage; Coef, coefficient; CI, confidence interval.

Table S10. RDW in the associations between n-3 blood biomarkers and 5-year mortality post-MI (KHB mediation analysis, not adjusted for CRP).

| Fatty Acid | Total Effect | |  | Direct Effect | |  | | Indirect Effect | | Indirect Effect/ |
| --- | --- | --- | --- | --- | --- | --- | --- | --- | --- | --- |
| biomarkers | Coef (95% CI) | *P* value | Coef (95% CI) | | *P* value | | Coef (95% CI) | | *P* value | Total Effect (%) |
| n-3FA | -0.416 (-1.063, 0.230) | 0.207 |  | -0.334 (-0.984, 0.315) | 0.313 |  | | -0.081 (-0.132, -0.031) | 0.001** | 19.67 |
| n-3FAp | -0.145 (-0.239, -0.051) | 0.002** |  | -0.135 (-0.229, -0.041) | 0.005** |  | | -0.009 (-0.015, -0.003) | 0.003** | 6.55# |
| DHA | -1.993 (-3.989, 0.003) | 0.051 |  | -1.649 (-3.614, 0.315) | 0.069 |  | | -0.134 (-0.225, -0.043) | 0.019* | 6.72 |
| DHAp | -0.288 (-0.488, -0.087) | 0.005** |  | -0.274 (-0.474, -0.073) | 0.007** |  | | -0.013 (-0.024, -0.003) | 0.013* | 4.83# |
| eEPAp | -0.201 (-0.344, -0.058) | 0.006** |  | -0.184 (-0.327, -0.041) | 0.011* |  | | -0.016 (-0.027, -0.006) | 0.002** | 8.29# |

** *p* < 0.01; * *p* < 0.05.

# *P* values for the total effect, direct effect, and indirect effect are all less than 0.05.

RDW, red cell distribution width - coefficient of variation; MI, myocardial infarction; CRP, C-reactive protein; n-3FA, omega-3 fatty acids; n-3FAp, omega-3 fatty acids to total fatty acids percentage; DHA, docosahexaenoic acid; DHAp, docosahexaenoic acid to total fatty acids percentage; eEPAp, estimated eicosapentaenoic acid to total fatty acids percentage; Coef, coefficient; CI, confidence interval.

Table S11. RDW in the associations between n-3 blood biomarkers and 5-year mortality post-MI (KHB mediation analysis, complete-case cohort, n = 3230, not adjusted for CRP).

| Fatty Acid | Total Effect | |  | Direct Effect | |  | | Indirect Effect | | Indirect Effect/ |
| --- | --- | --- | --- | --- | --- | --- | --- | --- | --- | --- |
| biomarkers | Coef (95% CI) | *P* value | Coef (95% CI) | | *P* value | | Coef (95% CI) | | *P* value | Total Effect (%) |
| n-3FA | -0.905 (-1.841, 0.031) | 0.058 |  | -0.843 (-1.817, 0.131) | 0.078 |  | | -0.062 (-0.084, -0.040) | 0.041* | 6.88 |
| n-3FAp | -0.225 (-0.261, -0.189) | 0.019* |  | -0.212 (-0.410, -0.014) | 0.032* |  | | -0.013 (-0.024, -0.002) | 0.043* | 5.89# |
| DHA | -2.511 (-5.216, 0.194) | 0.102 |  | -2.336 (-4.683, 0.011) | 0.061 |  | | -0.175 (-0.231, -0.119) | 0.020* | 6.97 |
| DHAp | -0.263 (-0.572, 0.046) | 0.079 |  | -0.252 (-0.492, -0.012) | 0.031* |  | | -0.011 (-0.029, 0.007) | 0.317 | 4.21 |
| eEPAp | -0.247 (-0.478, -0.016) | 0.028* |  | -0.226 (-0.413, -0.039) | 0.034* |  | | -0.021 (-0.040, -0.002) | 0.038* | 8.51# |

* *p* < 0.05.

# *P* values for the total effect, direct effect, and indirect effect are all less than 0.05.

RDW, red cell distribution width - coefficient of variation; MI, myocardial infarction; CRP, C-reactive protein; n-3FA, omega-3 fatty acids; n-3FAp, omega-3 fatty acids to total fatty acids percentage; DHA, docosahexaenoic acid; DHAp, docosahexaenoic acid to total fatty acids percentage; eEPAp, estimated eicosapentaenoic acid to total fatty acids percentage; Coef, coefficient; CI, confidence interval.

Figure S1


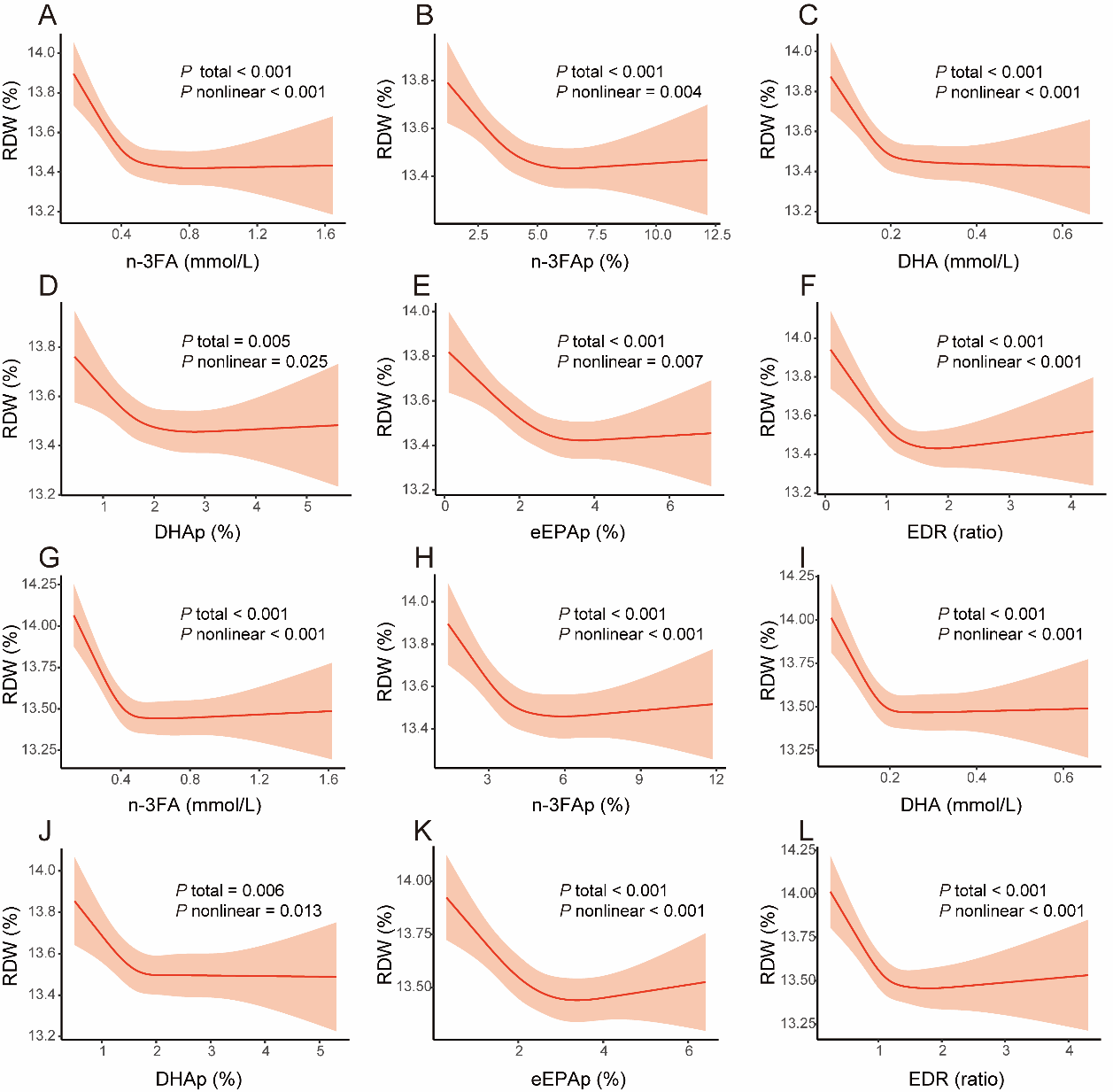


Figure S1. Associations between n-3 blood biomarkers and RDW: RCSs based on linear regression.

A to F show the associations in the original cohort (n=4043). G to L show the associations in the complete-case cohort (n=3230).

RDW, red cell distribution width - coefficient of variation; n-3FA, omega-3 fatty acids; n-3FAp, omega-3 fatty acids to total fatty acids percentage; DHA, docosahexaenoic acid; DHAp, docosahexaenoic acid to total fatty acids percentage; eEPAp, estimated eicosapentaenoic acid to total fatty acids percentage; EDR, the ratio of eEPAp to DHAp; RCS, restricted cubic spline.

Figure S2


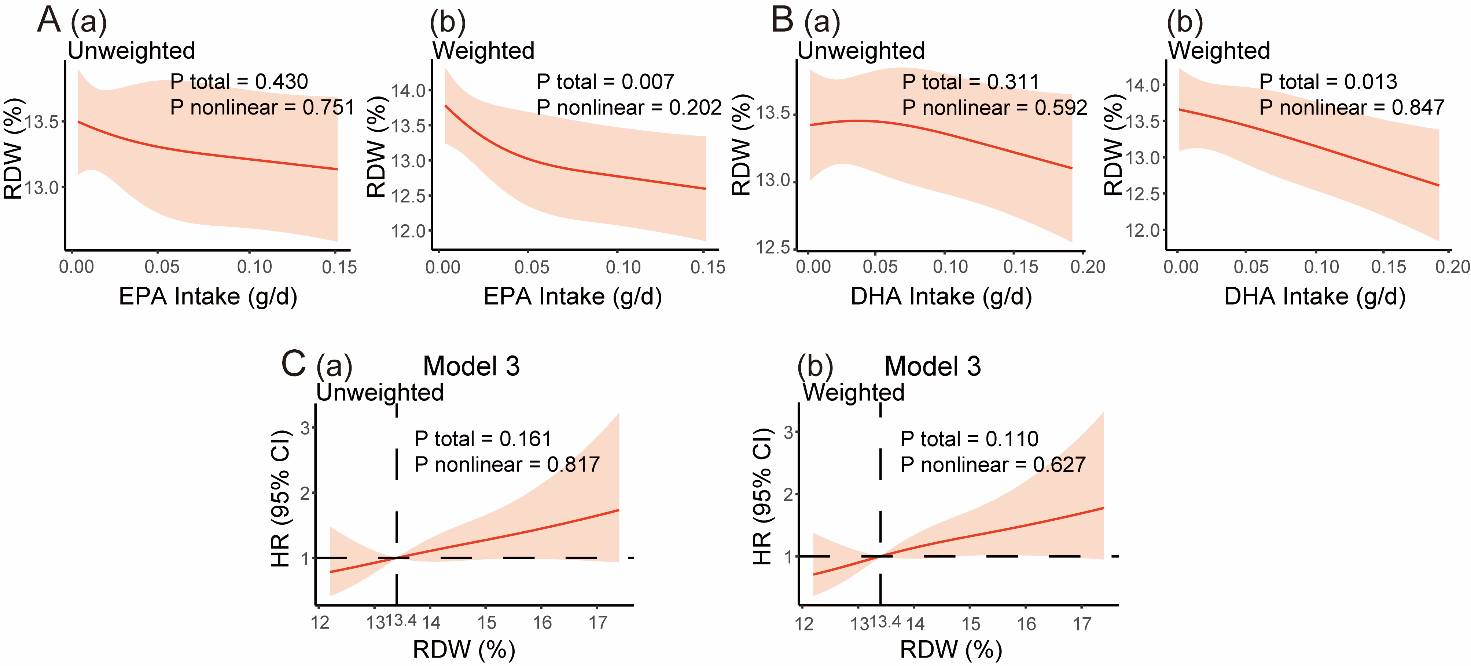


Figure S2. Adjusted associations between dietary n-3 FAs, RDW, and mortality.

Panels labeled (a) show the unweighted analyses, and those labeled (b) show the weighted analyses.

Model 3: further adjusted for monthly family income and diet score in addition to the covariates included in Model 2.

A and B. The RCSs based on linear regression show the associations between daily EPA and DHA intake and RDW. C. The RCSs based on Cox regression show the associations between RDW and mortality.

RDW, red cell distribution width - coefficient of variation; FA, fatty acid; EPA, eicosapentaenoic acid; DHA, docosahexaenoic acid; HR, Hazard Ratio; CI, confidence interval; RCS, restricted cubic spline.

Figure S3


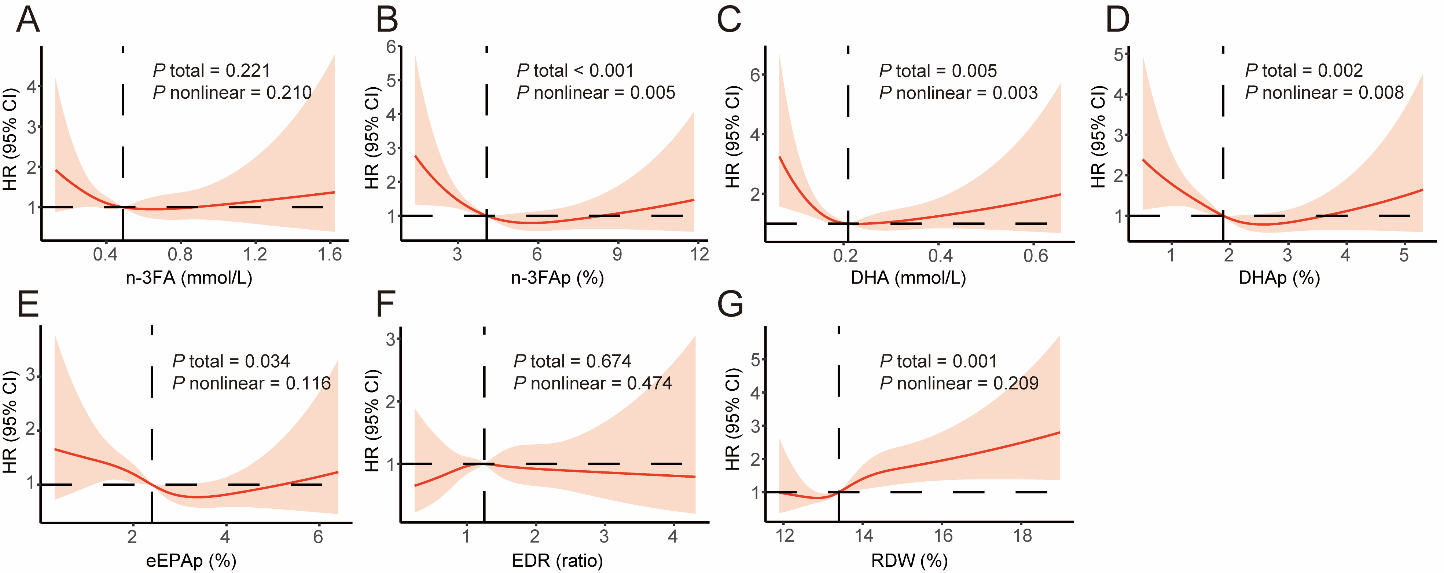


Figure S3. RCSs from Cox models of n-3 biomarkers and RDW on 5-year mortality (complete-case cohort, n=3230).

RDW, red cell distribution width - coefficient of variation; n-3FA, omega-3 fatty acids; n-3FAp, omega-3 fatty acids to total fatty acids percentage; DHA, docosahexaenoic acid; DHAp, docosahexaenoic acid to total fatty acids percentage; eEPAp, estimated eicosapentaenoic acid to total fatty acids percentage; EDR, the ratio of eEPAp to DHAp; HR, Hazard Ratio; CI, confidence interval; RCS, restricted cubic spline.
